# Multilayer Bixin Microcapsules: The Impact of Native Carbohydrates on the Microencapsulation Efficiency and Dispersion Stability

**DOI:** 10.3390/foods8030108

**Published:** 2019-03-22

**Authors:** Cecilia K. Curi-Borda, Javier A. Linares-Pastén, Tuba Tat, Rosmery Tarqui-Dueñas, Ninoska Chino-Flores, Juan-Antonio Alvarado, Bjorn Bergenstahl

**Affiliations:** 1 Food Science and Technology Department, Lund University, P.O. Box 124, SE-22100 Lund, Sweden; 2 Institute of Chemical Research, Universidad Mayor de San Andrés, 27th Cota Cota, P.O. Box 303, La Paz, Bolivia; tarqui.rosmery.a@gmail.com (R.T.-D.); ninoskachino@gmail.com (N.C.-F.); jaalvkir@gmail.com (J.-A.A.); 3 Division of Biotechnology, Department of Chemistry, Lund University, P.O. Box 124, SE-22100 Lund, Sweden; javier.linares-pasten@biotek.lu.se; 4 Department of Food Engineering, Ankara University, TR 06532 Ankara, Turkey; ttat@ankara.edu.tr

**Keywords:** bixin, hemicellulose, starch, spray drying, encapsulation efficiency, aqueous dispersion, physical stability

## Abstract

Bixin is a hydrophobic carotenoid present in the integument of the seeds of *Bixa orellana*. Microencapsulation was applied to obtain water dispersible formulations and protect the colorant against degradation. Microencapsulated systems were obtained by spray-drying a mild alkaline bixin dispersion with different encapsulating materials. The encapsulation trials were performed with and without native carbohydrates of the integument in addition to the main encapsulant. It was possible to dry dispersions with up to 10% bixin counted on total solids. All the studied systems were characterized by colorimetry, UV-vis spectroscopy, Scanning Electron Microscopy, light microscopy, turbidometric sedimentation analyses and laser light diffraction analyses. All the systems showed aqueous dispersibility but displayed differences in their transparency, UV-vis spectra and physical stability at pH 3. The results show that the native carbohydrates enhance the encapsulation efficiency of other encapsulating materials. The chemical composition of this native carbohydrate fraction shows the presence of polysaccharides containing arabinose, galactose and glucose as monomers. Starch was identified enzymatically. The native carbohydrates allowed the encapsulation of bixin in its native microcrystalline form, resulting in a multilayer structure after spray-drying. In addition, the colorant particles displayed dispersibility under acidic aqueous conditions suggesting that they are stabilized by the native carbohydrates after the microcapsules are dissolved.

## 1. Introduction

During the last 20 years, the demand for natural pigments for coloring foods has increased because of regulatory limitations to the usage of synthetic colorants, as well as increasing consumer interest in more natural alternatives [1]. Attention has been drawn to *Bixa orellana*, a shrub cultivated in countries such as Mexico, Brazil, Bolivia, Colombia and Peru in Latin America, as well as India in Asia among others [2,3,4]. *Bixa orellana* is known by different names as achiote (Spanish), annatto (English) and urucum (Portuguese) [5]. The colored extract called by the same name “annatto” is used for coloring different foods. Nowadays, annatto as a food coloring additive has become one of the highest globally traded natural colorants with approximately 7500 tons of seeds annually imported by the USA, Western Europe [6] and Japan [3,5]. 

The intense orange to red colored waxy integument layer that surrounds the black annatto seeds contains two major carotenoids that are responsible for the color: 9′-cis-bixin (bixin) and 9′-cis-norbixin (norbixin). The sclerified palisade cell layer that constitutes the integument is composed of hemicellulose, starch and a small amount of protein and ash in addition to the pigment [7]. Several methods have been applied to extract these components and the carotenoids from the seed integument [2,4,8,9]. 

**Figure 1:**
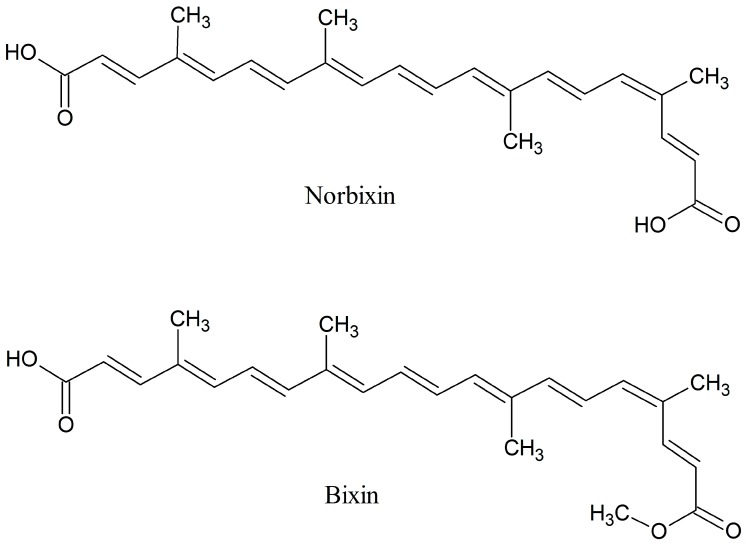
Chemical Structures of bixin and norbixin.

Within the integument, bixin represents 80% of the existing colorants followed by cis-norbixin, several other carotenoids [9] and phenolic compounds [10]. Bixin and norbixin are lipophilic colorants, practically insoluble in water, and somewhat soluble in methanol and other solvents of intermediate polarity like dichloromethane, ethyl acetate, acetone and acetonitrile [9,11,12]. Bixin is the monomethylester of the dicarboxylic acid norbixin (Figure 1). It can be hydrolyzed to norbixin salts under alkaline conditions [12]. These salts remain water soluble at alkali pH but will precipitate under acidic pH as the dicarboxylic acid is formed. 

Due to the physicochemical characteristics, annatto extracts and the isolated carotenoids are commonly used in fat rich foods including cheese, emulsion, snacks, desserts, cakes, ice cream, spreads, fillings and margarine [2,12,13,14,15]. However, the application is limited by its sensitivity to oxidation, degradation when exposed to high temperature and light, and its lack of solubility in water [12]. Thus, there is a need for water dispersible natural colorant formulations that are stable towards light and temperature to expand the use of these carotenoids in acidic aqueous food products. Several techniques have been developed to obtain water dispersible formulations, which can also protect the capacity of the carotenoids. Some of them are aimed at the extension of the shelf life of the colorant, and include the addition of antioxidants [16], as well as the modification of extraction processes and atmosphere [12]. Studies on water dispersible formulations include the addition of emulsifiers and the creation of microencapsulated formulations [17]. Microencapsulation is a convenient technique for improving carotenoid stability, and for modifying the properties of the pigment formulation to enable water dispersibility of the carotenoids [17].

The microencapsulation of bixin or norbixin can be performed by either spray-drying or freeze-drying a dispersion of the colorant particles in a solution of the encapsulating material (e.g., saccharides, polysaccharides, proteins). Encapsulation using maltodextrin and sucrose has allowed an increased light stability [18,19]. Another study has proved that bixin encapsulated with sodium caseinate had a higher thermal stability than non-encapsulated bixin [20]. 

The spray-drying process for encapsulation includes several steps as follows. The first step is to create a dispersion of a target substance (core) in a solution of low molecular weight molecules or polymers that may act as the surrounding material (wall material) protecting the encapsulated core. Then, the dispersion (feed) is pumped into the spray-dryer. The feed passes through a nozzle and is atomized into a chamber and heated by a co-current flow of hot air. The small droplets are dried by the hot air flow inside the chamber in less than a second. While the water is being evaporated, the wall material builds up a capsule enclosing the hydrophobic compound [17]. 

The selection of the wall material is critical for increasing the encapsulation efficiency of the core, and thus, decreasing its exposure to light and environment. The most common wall materials are saccharides, polysaccharides, gums and proteins [17]. Hemicelluloses are carbohydrates worth considering as wall materials because of their thickening and stabilizing properties [21,22]. Other important properties include film forming and emulsifying properties [23,24]. However, there are few studies about the use of hemicelluloses as encapsulant materials. Recent studies include the use of hemicelluloses from corn for the encapsulation and stabilization of flavors [21], and fish oil [25]. 

The hydrophobic compound present in the microcapsule core is dispersed in water once the wall material of the microcapsule is dissolved. Therefore, the physical stability of the hydrophobic compound aqueous dispersion will be affected by several factors. Some of them include the particle size distribution, rheology, environmental conditions, and the repulsive or attractive interactions between particles [26]. The interrelation of these factors produces several instability mechanisms such as sedimentation or creaming, and flocculation or coalescence of the hydrophobic particles [27]. 

The structural properties of bixin could be used for obtaining aqueous dispersions that can be fed into the spray-dryer. Consequently, the purpose of the present study was to apply spray-drying as a technique to obtain water dispersible bixin microcapsules. Furthermore, we proposed to evaluate the microencapsulation efficiency of different mixtures of wall materials, and assess the physical stability of the colorant particles in aqueous dispersions for their use in aqueous products. Moreover, to the best of our knowledge, there are no evaluations of the role of the native carbohydrates of *Bixa orellana* seed integument as an encapsulant material, or as an encapsulant enhancer. The native carbohydrates present in the annatto extracts obtained by abrasion or other processes, may contribute to the encapsulation efficiency or dispersion stability of the colorant. Thus, the native carbohydrates were characterized, as well as its contribution to the encapsulation efficiency and the product properties. 

## 2. Materials and Methods

### 2.1. Materials

Bixin was extracted and purified from the seeds of *Bixa orellana* obtained from a local market in La Paz city, Bolivia. Maltodextrin (MD), DE23.5 was purchased from Equal International Corporation (Shanghai, China). Gum arabic from the acacia tree, a branched polymer of galactose, rhamnose, arabinose and glucuronic acid, with a molecular weight of approximate 250,000, was purchased from Sigma Aldrich (St. Louis, MO, USA). Carboxymethylcelullose (CMC), DS 0.79 was obtained from Amtex (Medellin, Colombia). Citric pectin, obtained mainly of the rind of citrus peels, with a degree of esterification of 65%, medium rapid set, and gel strength of 150 at 1% solution at pH 3.6 according to USA-SAG method, was purchased from CP Kelco (São Paulo, Brasil). Sweet whey powder (13% protein, 78% lactose, 0.7% lipids, 7% Ash, pH~6) was purchased from Colun Ltd. (La Unión, Chile). Sucrose (>99.5% purity) was purchased from Guabirá S.A. (Santa Cruz, Bolivia).

Methanol, petroleum ether (40–60 °C), ethyl acetate and ethanol used for bixin extraction were commercial grade. All the analyses were performed using analytical grade dicloromethane (Merck, Darmstadt, Germany), acetonitrile (Avantor, Center Valley, PA, USA) and methanol (Sigma Aldrich, St Louis, MO, USA). For all experiments where water was required, deionized and purified water was obtained using MiliQ system, (Merck Millipore, Darmstadt, Germany).

### 2.2. Colorant Extraction

For all experiments bixin, with or without the naturally present carbohydrates from the integument, was used as the coloring agent. The coloring agents were obtained with a modified version of the method reported by Rios & Mercadante [28]. *Bixa orellana* coated seeds were first washed three times with petroleum ether followed by a wash with methanol using a 1:2 solid-solvent ratio for the removal of nonpolar and polar compounds respectively. Thereafter, the colored integument was extracted (suspended) in ethyl acetate (2:1 solid-solvent ratio), and this suspension of integument particles was collected after a 500 μm sieve to remove the seeds. The colored integument was extracted until the seeds were colorless. After this, the integument particles were recovered after removing the solvent phase by filtering through a 40–60 μm pore size glass frit. The solid filter-cake was washed with a mixture of 1:4 dichloromethane-ethanol for further purification and finally dried and stored in darkness at ambient conditions. The dried filter-cake material is named “Carbohydrate rich bixin—CRB” and is composed of bixin and the native polysaccharides present in the seed integument. The CRB was ground with a mortar. 

Purified bixin crystals (PB) were extracted from the CRB extract by dissolving bixin in a large surplus of ethyl acetate and collecting the filtrate after filtrating it through a 10–20 μm glass fritted funnel into a round bottom flask for final drying in a rotaevaporator at 30 °C. The CRB extract and PB presented a peak area of 97% in the HPLC Chromatogram. The HPLC method used was the one reported by Rios & Mercadante [28] with acetonitrile −0.01% acetic acid as the mobile phase. 

PB was selected as a coloring agent because of the need for more concentrated pigments for coloring food products. However, the present work aims to asses if keeping the native carbohydrates of the CRB extract could improve the value of bixin as a coloring agent. A higher value of a coloring agent could be traduced in an improvement of the colorant dispersibility in water, and an enhancement of the encapsulation efficiency of bixin.

### 2.3. PB and/or CRB Suspensions

Two sets of dispersions were used for the encapsulation experiments using spray drying. Both consisted of water suspensions of PB or CRB extract dispersed in a solution of an encapsulating agent. The suspensions were made by taking PB or CRB extract and adding sodium carbonate in a dry state. The PB to carbonate mass ratio was approximately 4:1 and the CRB to carbonate mass ratio was 5:1. This relation was calculated stoichiometrically assuming a 1:1 molar ratio. The systems were mixed with water gradually and homogenized for 3 min at 15,000 rpm with a 6V shaft (Unidrive X-1000, CAT Scientific, Paso Robles, CA, USA). The encapsulating agent was dissolved separately in water at 70 °C, stirred until it was homogenous and cooled to 30 °C. After that, the encapsulating agent solution was added to the PB or CRB suspension and mixed together. The volume was adjusted with water to obtain 1.0% of PB, or 2.5% of CRB (*m*/*v*), and 10% *w*/*v* of total solids. The composition of total solids of the different suspended systems prepared for spray-drying is presented in Table 1. The final suspension was homogenized for 2 min 15,000 rpm. 

The proportions of maltodextrine, gum arabic, CMC and pectin were selected based on previous studies on microencapsulation [29] with the purpose of having an appropriate density and flowability for introducing the suspension into the spray-dryer. Preliminary tests were carried out, preparing mixtures of micoencapulant materials at different concentrations. 

For the last system shown in Table 1, the CRB extract was suspended in water with sodium carbonate, filled up to a final volume of 10% *w*/*v*, homogenized, and spray-dried. The obtained spray-dried powder was named CRB-encap.

### 2.4. Spray Drying of the Suspensions

The co-current lab spray-dryer YC-015, Shanghai Y. Instruments Co. Ltd., Shanghai, China, with two-fluid jet nozzles for atomization, was used for spray-drying the different suspensions. The spray drier was operated at a liquid flow rate of 7 mL/min. The spray drying inlet temperature was set to 120 °C and the outlet temperature was between 54–59 °C. The air pressure was 2–3 bar and an aspersion nozzle diameter of 1 mm. Every encapsulated system was done in duplicates (Replicate (1) & (2)). An even concentration of the feed during spray drying was secured by stirring. The water activity of all the microencapsulated powders was measured using a Rotronic Hygropalm HP23-A multifunction hand held indicator (Rotronic, Bassersdorf, Switzerland). 

### 2.5. Bixin Spectrophotometrical Analysis

**Figure A1:**
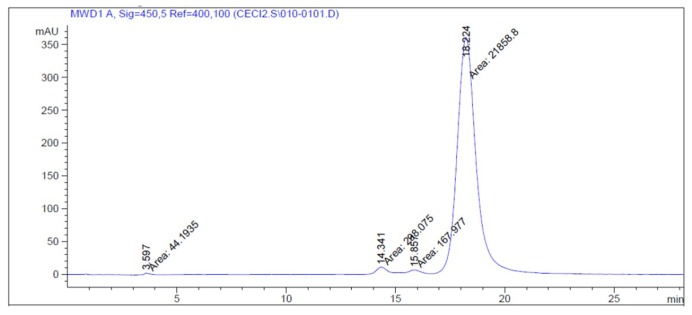
Cromatogram of Bixin after extraction.

Bixin was analyzed spectrophotometrically at 468 nm in dichloromethane. The extinction coefficient used was $E_{1cm}^{1\%}=2810$. This value was obtained from a calibration curve in dichloromethane. The UV-vis spectrum of bixin is shown in Appendix A, Figure A1. 

#### 2.5.1. Total Bixin Determination

Total bixin was determined by dissolving 100 mg of microencapsulated powder in 6 mL of water. The solution/dispersion was transferred to a separation flask where it was mixed with 2.5 mL of methanol followed by the addition of 20 mL of dichloromethane. The two phases were exhaustively agitated to extract the bixin into the organic phase. After the two phases were separated, the organic phase was collected, filtered using a 0.22 μm pore size PTPF filter, diluted and quantified by spectrophotometry at 468 nm. A second extraction of bixin was performed by adding another 20 mL of dichloromethane after the first extraction and agitating until no color was observed in the aqueous phase. Total bixin determination was done in duplicate for each sample. 

#### 2.5.2. Surface Bixin Determination

It is assumed that some of the bixin is located at the surface of the microcapsules. The surface bixin is assumed to be the rapidly extractable bixin. Exactly 20 mg of encapsulated powder was dispersed with 10 mL of dichloromethane in a vortex for 30 s followed by immediate filtration using a 0.22 μm pore size PTPF filter. After filtration, the sample was diluted and quantified using a Perkin Elmer, Lambda 25 spectrophotometer, at 468 nm. The surface bixin determination was done in triplicate for each sample. 

#### 2.5.3. Encapsulation Efficiency Measurements

The extinction coefficient was used for calculating all the encapsulation efficiencies and bixin contents according to Beer’s law (See Supplementary Materials). The encapsulation efficiency *EE* was measured by Equation (1) as proposed by Barbosa [18]:

(1) $$EE=\;\frac{\left(T_{B}-S_{B}\right)}{T_{B}}$$

where *T_B_* is the total bixin and *S_B_* is the surface bixin.

### 2.6. Microcapsules Characterization:

#### 2.6.1. Particle Size Distribution

The particle size distribution of the PB or the CRB microcapsules was measured by laser diffraction using a Malvern particle size analyzer, Mastersizer 2000S, Malvern Instruments Ltd., Malvern, UK. Microcapsules were dissolved/dispersed in Milli-Q water until the obscuration ranged between 5% and 10%. The colorant dispersion was pumped through the optical chamber at 2000 rpm. The refractive index (RI) used for the dispersed phase was 1.52 and the RI used for pure water was 1.33. 

A Malvern particle size analyzer, Zetasizer Nano series, Malvern Instruments Ltd., Malvern, UK was used for analyzing particle size distribution of colorant particles dispersed in water. The samples were placed in polystyrene cuvettes of 1 cm length. Also, this equipment was used for measuring the zeta-potential of the colorant particles dispersed in water. The samples were placed in a folded capillary cell DTS1060. After 40 days of the physical stability study of the microcapsules dissolved in water (Section 2.7.2), the particle size distribution of the colorant particles present in the supernatant of all the dispersions was measured with the Zetasizer. Zeta-potential was measured on the samples that had particles remaining in the supernatant after the 40 days. 

#### 2.6.2. Scanning Electron Microscopy

The morphology of all the microencapsulated powders was assessed by taking images of each system with a JEOL JSM-6700F, JEOL, Tokyo, Japan, scanning electron microscope (SEM), set to an acceleration voltage of 10 kV. Lower detection imaging mode (LEI) was used to get the images of the sample surface at variable distances around 8 mm. The samples were prepared by depositing a small amount of the microencapsulated powder of each system on a brass stub with sticky tape. The sticky tape and sample were then covered with a 15 nm layer of Au/Pd using a Balzer sputter coater SCD004. 

#### 2.6.3. Optical Microscopy

Each of the microencapsulated powders and their respective water dispersions (Section 2.7) were observed under a light Microscope Olympus BX50, Tokyo, Japan. The magnification of the objective varied between 20–100×. The objectives used were 20×/0.4 ∞/0 LMPlanFl, 50×/0.5 ∞/0 LMPlanFl and 100×/1.25 Plan (immersion oil). Images were taken with a digital camera DFK 41AF02, Imaging Source, Germany, and processed with the software ImageJ v1.42 m, W. Rasband, National Institute of Mental Health, Bethesda, MD. Microcapsules were placed on a microscope slide and dispersed in MCT oil. 

### 2.7. Characterization of Water Dissolved Microcapsules

#### 2.7.1. Colorimetric Analysis

The color of the microcapsules dissolved/dispersed in water was determined using CIE Lab space, where the CIE Lab parameters a, b and L were obtained using a Konica Minolta Spectrophotometer CM-700 d/600 d. Samples were prepared by dissolving/dispersing 1.5 mg/mL of Bixin microcapsules, 0.3 mg/mL of CRB microcapsules or 0.048 mg/mL of CRB-encap in water. Each sample was placed in an open Petri dish on top of a white surface.

UV-visible spectra were taken using a UV/Vis Spectrophotometer PerkinElmer, Lambda 25, from 350 nm to 600 nm. The spectra were taken before (0 h) and after (16 days or 40 days) the physical stability tests (Section 2.7.2). Initially, microcapsules were dissolved in water and introduced in the spectrophotometer. After the stability tests, the visible spectrum of the supernatant of each sample was measured. Concentrations of 0.3 mg/mL (CRB-microcapsules, CRB and CRB-encap) and 1.5 mg/mL (PB-microcapsules) were used. Since the initial concentration of CRB and CRB-encap was too high for spectrophotometric measurements, UV-Visible spectra were taken at concentrations of 0.1 mg/mL and 0.048 mg/mL for CRB and CRB-encap respectively for 0 h. 

A turbidity measurement was made by the method reported by Balch [30]. A comparison between samples was assessed at 0 h by comparing the absorbance of the samples at 600 nm concerning pure water. The concentrations used were the same as the ones for 0 h. 

#### 2.7.2. Physical Stability of the Dispersions

The physical stability of the dispersions was monitored by light scattering using the Turbiscan LAB, Formulaction SAS, L’Union, France. The apparatus has an electro luminescent diode as a light source that emits light at 880 nm. Two sensors, positioned at 180° and 45° from the light source, detect transmitted, or back scattered light, respectively. The Turbiscan was operated at Backscattering and Transmission mode, scanning from top to bottom, every 40 μm of the sample length. The frequency of each scan started with every minute for the first hour, followed by scans every hour for the first 24 h, and by scans every 2–5 days for 40 days. The working temperature was 25 °C. Replicates (1) of the samples were prepared by dissolving in water 0.3 mg/mL of CRB, CRB-encap, CRB-microcapsules and PB-microcapsules. Replicates (2) of samples PB-MD and PB-MD-GA were prepared at 1.5 g/mL. A few drops of HCl 0.2 N were added to adjust to pH 3. Turbisoft Lab 2.2.0.82-5 software was used to analyze the collected data. 

The physical instability of a sample for a specific time *t*, was assessed by the change (relative to time zero) of transmittance as a function of time and position: $\Delta \tau \;\left(h_{i,t}\right)\;=\;\left|\tau \left(h_{i,t}\right)\;–{\;\tau }_{0}\left(h_{i}\right)\right|$. Where $\tau_{0}\left(h_{i}\right)$ is the transmittance at time 0 and height (*h*), and $\tau \left(h_{i,t}\right)$. is the transmittance at height (*h*) and time (*t*).

The profile of the scans is characteristic for identifying the instability mechanism as sedimentation (creaming), flocculation or coalescence. The Turbiscan Stability Index (*TSI*), which shows that as higher the value, as stronger the instability of the sample for a specific time, was computed by the following equation (Equation (2)):

(2) $$TSI\left(t\right)=\frac{\sum_{i=1}^{n}\Delta \tau_{i}\left(h_{i},t\right)}{n}$$

where *n* is the number of measurement levels.

### 2.8. Chemical Analysis of the Annatto Seed Integument Carbohydrate Fraction

#### 2.8.1. Enzymatic Analysis

Specific endo-glycoside hydrolases were used to identify polysaccharides. Endo-1,5-α-arabinase from *Aspergillus niger* and cellulase from *Thermobifida halotolerans* were purchased form Megazyme (article numbers E-EARAB and E-CELTH respectively, Wicklow, Ireland), α-amylase was purchased from Sigma Aldrich (St. Louis, MO, USA). The reactions were performed at pH and temperatures recommended by the manufacturers. Xylan determination was performed with β-1,4-xylanase such as is described in Gil-Ramirez et al. [31]. The CRB sample concentration was 1% *w*/*v* in all enzymatic reactions. The products were determined after 14 h with DNS method for reducing end sugars as is described in Faryar et al. 2015 [32]. 

#### 2.8.2. Analysis of Monosaccharide Composition of the Integument

The CRB was extracted directly from the seeds of *Bixa orellana* by solid-liquid extraction; 10 g of seeds were weighted in a flask. Then, between seven and ten aqueous extractions were performed. For each extraction, a solid portion of approximately 0.015 g of sodium carbonate was added to the seeds and mixed together. Subsequently, near 5 mL of water were added gradually to suspend the integument particles. The suspension was transferred to a 100 mL volumetric flask. The seeds were rinsed twice with a total of 5 mL, which was transferred as well to the flask; all this before the next extraction. The seeds integument was extracted until the seeds were colorless. Then, sodium carbonate was added to the suspension to reach a final amount of 0.15 g in the volumetric flask, and the aqueous suspension was filled up to the final volume of 100 mL. After this, the suspension was transferred to a beaker, and the pH of the suspension was adjusted to pH 3 by adding a few drops of HCl 0.2 N. After some days, the suspension settled and the two-phase system was freeze dried in a freeze drier Labconco (Kansas City, MO, USA).

Ten milligrams of dried samples of the CRB extract were subjected to Seaman Hydrolysis. Initially, the samples were hydrolyzed with 72% sulfuric acid for 60 min at 30 °C. After, the reactions were diluted to 4% *v*/*v* with deionized water and incubated at 100 °C for 3 h. Then, the reactions were cooled down at room temperature. An aliquot of 1 mL was taken for neutralization with 0.1 M Ba(OH)_2_.H_2_O to a pH between 5 and 6. After that, the samples were filtered with a 0.22 μm membrane filter, diluted 20 times with ultra-pure water, and analyzed with high-performance anion exchange chromatography with pulse amperometric detectior (HPAEC-PAD) (Sunnyvale, CA, USA). A PA-20 column was used with NaOH as the mobile phase, as described in [31,33]. Standards of glucose, galactose, manose, xylose, and arabinose were prepared in concentration from 0.5 to 20 μg/mL.

## 3. Results

### 3.1. Bixin and CRB Purity

**Figure A2:**
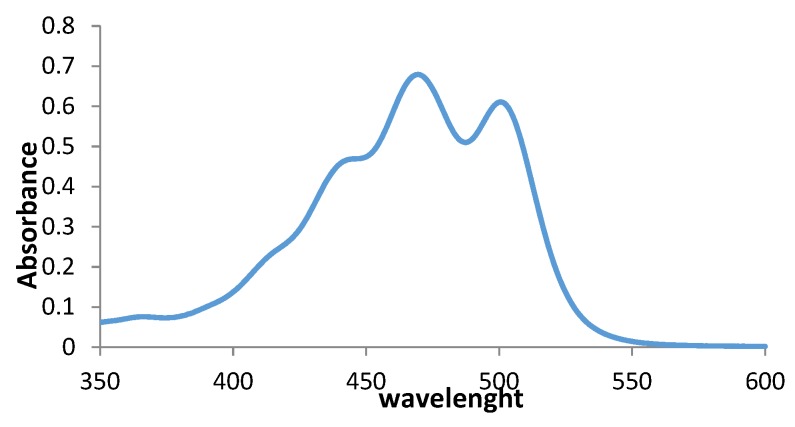
Bixin UV-vis spectrum in Dicloromethane (3 ppm), after extraction.

Pure bixin crystals (PB) and Carbohydrate rich bixin (CRB) extract obtained by solvent extraction from the seed coat were analyzed using a reversed phase HPLC column. A single peak was observed with a retention time of 26 min (Appendix A, Figure A2) comparable to the results of Rios & Mercadante [28]. The area of the chromatogram peak corresponded to 97% of the total area of the chromatogram. This value is comparable to the ones reported by Rios and Mercadante (98%) [28], Barbosa et al. (94%) [18] and Lobato et al. (98.7%) [34] Also, the absorption spectrum was characteristic for bixin (Appendix A). Hence, the identity was confirmed and the extraction procedure was considered successful for obtaining high purity bixin in both cases.

### 3.2. Bixin and CRB Suspensions

PB crystals and CRB extract were dispersed in water using mild alkaline conditions with sodium carbonate (<0.4% *w*/*v*), creating intense orange-redish suspensions. The suspensions appeared stable although large particles were settling. To avoid sedimentation, stirring was used for securing a homogeneous feed while the samples were spray dried. Nachtigall and Silva have reported that no saponification of bixin is observed when the concentration of base was under 0.4% *w*/*v* [35,36]. In our samples, this was verified by comparing the retention time of the bixin’s peak in HPLC with the corresponding peaks of the spray dried samples. The mild alkaline conditions allowed us to have a spray drying feed of 1.2% *w*/*v* bixin suspensions obtaining a spray dried powders of 10% *w*/*w* bixin counted on total solids. After spray-drying, the microcapsules dispersed in water showed pH values between 6.5 and 9. 

### 3.3. Encapsulation Result and Efficiency

Total bixin present in the microcapsules as well as the water activity of each microencapsulated system is reported in Table 2. The variations observed between the total bixin in the spray dried microcapsules and the initially formulated bixin dispersion (10% bixin before spray drying) is most likely because of loss of material during the spray drying, resulting in recoveries of around 50% of total spray dried solids. 

**Figure 2:**
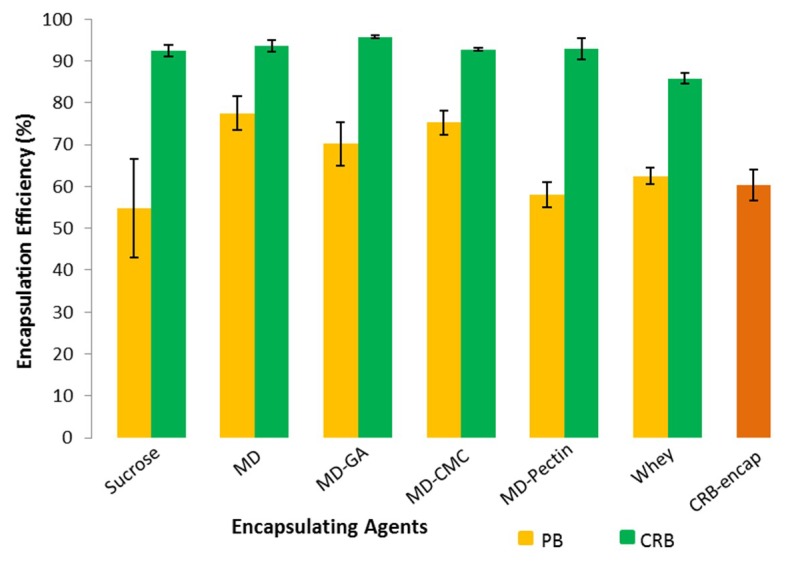
Encapsulation efficiencies for pure bixin crystals (PB) or carbohydrates rich bixin (CRB) extract spray dried with different wall materials. Error bars represent the standard deviation (*n* = 6). (MD: Maltodextrin, GA: Gum arabic, CMC: Carboxymethylcellulose, CRB-encap: Spray dried CRB).

The PB crystals or CRB extract were encapsulated by spray drying the aqueous pigment suspension together with a dissolved encapsulating material. The encapsulating material may consist of just carbohydrates (sucrose or maltodextrin), whey protein or a carbohydrate together with a surface active encapsulating enhancing material (gum arabic, pectin, carboxymethylcellulose). After spray-drying, the encapsulation efficiency was measured for each system. These values are presented in Figure 2.

ANOVA statistical analyses of the encapsulation efficiencies (95% confidence) for pure bixin systems, indicate that the lowest protection of PB is given by sucrose as an encapsulant material (*p* < 0.05). Better encapsulation efficiency is given by CRB-encap, PB-MD-Pectin or PB-Whey with no significant differences between them (*p* > 0.05). The best protection of PB is observed when the encapsulant material is changed to only maltodextrin (*p* < 0.05). Similar values to MD are obtained when CMC (*p* > 0.05) and GA (*p* < 0.05) are added to MD. 

Interestingly, spray drying different wall materials together with the complex of bixin and the native carbohydrate fraction, resulted in increased bixin encapsulation efficiency. These results were above 90%, except for whey that was around 85%. The best encapsulating material systems were CRB-MD-GA and CRB-MD-Pectin, with no significant differences between them (*p* > 0.05). Significantly lower encapsulation efficiencies were obtained for CRB-MD and CRB-Sucrose, although no significant difference was observed compared to CRB-MD-Pectin. These systems were followed by CRB-MD-CMC, which had a lower encapsulation efficiency (*p* < 0.05). The lowest protection was given by CRB-Whey (*p* < 0.05). 

### 3.4. Microcapsules Characteristics

#### 3.4.1. Particle Size Distribution

After Spray drying, the microcapsules were dissolved in water, and the particle size distribution of the dispersed colorant particles was measured by light scattering. The values of volume weighted average diameter D_4,3_ are shown in Table 2. The volume weighted diameter distribution was bimodal for all the CRB encapsulated systems. The bimodal distribution consists of a narrow distribution from 0.05 μm to 0.5 μm and a second wide distribution between 1 μm and 100 μm. On the other hand, unimodal particle volume diameter distributions between 1 μm and 100 μm were observed for PB encapsulated systems. Yet, PB-sucrose, one of the replicas of PB-MD as well as of one replica of PB-MD-GA had bimodal distributions similar to CRB microcapsules. For CRB aqueous dispersions, unimodal distributions from 1 μm and 100 μm were obtained. It is important to mention that CRB had a D_0,1_ proximate to 7 μm. 

After 40 days of the physical stability tests (Section 3.5.2), smaller particles remained dispersed in the CRB samples. The volume weighted average diameter of the supernatants was measured by light scattering ($D_{4,3}^{40\;days}$). Samples containing CRB extract presented particle sizes between 0.2 μm and 0.8 μm (Table 2). The PB dispersions did not met quality criteria for measuring their particle size after 40 days, which was interpreted as the concentration was too low to measure. Surprisingly, no particles were detected either for CRB-Whey supernatant.

#### 3.4.2. Morphological Characteristics by SEM

**Figure 3:**
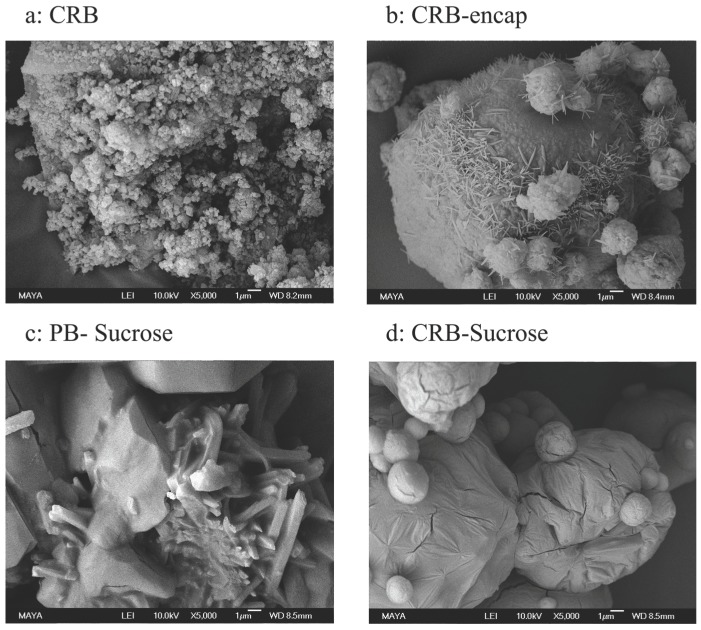
SEM images of (**a**) CRB: Carbohydrates rich bixin extract prior spray drying; (**b**) CRB-encap: Microcapsules of spray dried CRB extract; (**c**) CRB-Sucrose: Microcapsules of spray dried CRB extract together with sucrose; (**d**) PB-Sucrose: Spray dried pure bixin together with sucrose.

Scanning electron microscopy images were obtained for CRB and for the spray-dried systems. Figure 3a shows the native bixin integument as extracted from the plant, here named as carbohydrate rich bixin (CRB). The material appears as small particles (~1 μm) that are agglomerated into amorphous bigger aggregates (D_4,3_~70 μm). When bixin is extracted with the native carbohydrate fraction (CRB extract) followed by spray-drying, the native carbohydrates become the encapsulating agent. Then, spherical microcapsules are formed (CRB-encap) as shown in Figure 3b. However, the native carbohydrates by themselves display low encapsulation efficiency (i.e., 60%) as bixin crystals (~1 μm) appear at the surface of the capsules. 

Figure 3c shows how sucrose crystalizes along with PB after being spray dried, with no microcapsules formed as a consequence. Interestingly, spray drying bixin with a mixture of native carbohydrates and sucrose (CRB-sucrose) turns out to be a very effective encapsulant, with a 92% encapsulation efficiency, producing smooth sphere microcapsules (Figure 3d).

**Figure 4:**
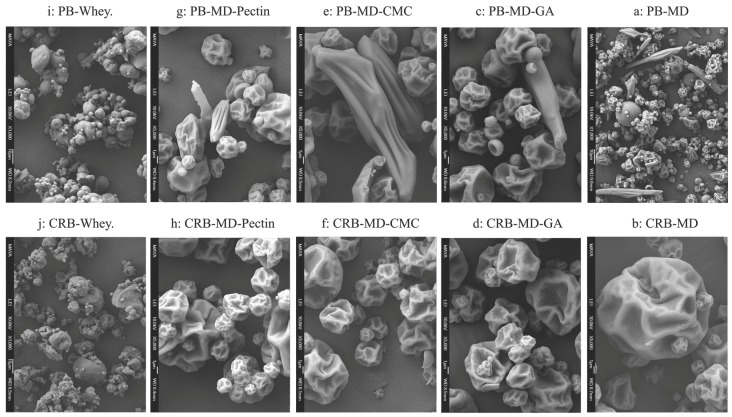
SEM images of Spray Dried bixin microcapsules using different wall materials. Left column corresponds to PB (bixin crystals) microcapsules whereas right column shows CRB (Carbohydrate rich bixin extract) microcapsules. Non-encapsulated colorant crystals can be observed when the native carbohydrates of the annatto extract are absent. Systems contain: (**a**) PB-maltodextrin, (**b**) CRB-maltodextrin, (**c**) PB-maltodextrin-gum arabic, (**d**) CRB-maltodextrin-gum arabic, (**e**) PB-maltodextrin-carboxylmethylcecllulose, (**f**) CRB-maltodextrine-carboxylmethylcecllulose, (**g**) PB-maltodextrine-pectin, (**h**) CRB-maltodextrine-pectin, (**i**) PB-Whey, (**j**) CRB-Whey.

When PB or CRB is encapsulated with a higher molecular weight oligosaccharide such as maltodextrin, spherical microcapsules with dents and ridges were formed in both cases. Figure 4a shows the microcapsules obtained from spray drying PB with maltodextrin. Notice the presence of bixin crystals that are not encapsulated, and have in most cases, bigger particle sizes than the average size of the microcapsules. On the other hand, CRB-MD (Figure 4b) did not present unprotected bixin crystals, which agree with the high encapsulation efficiency for this system. 

Encapsulating enhancing material such as gum arabic, pectin or carboxymethylcellulose was added to the maltodextrin (MD) to form an encapsulating mixture. Dented spherical particles with ridges were formed, similarly to what was observed for pure MD. Notice that the mixture of PB-MD-CMC produced elongated particles (Figure 4e). This could explain the higher encapsulation efficiency of PB-MD-CMC compared to PB-MD-GA (Figure 4c) and PB-MD-Pectin (Figure 4g). Pure bixin (PB) systems showed unprotected bixin crystals in contrast to CRB systems: CRB-MD-GA (Figure 4d), CRB-MD-CMC (Figure 4f) and CRB-MD-Pectin (Figure 4h). 

Spray-drying PB or CRB with whey produced agglomerates of fairly smooth spherical particles. Figure 4i shows PB-Whey microcapsules. Bixin crystals contained inside individual capsules agglomerate to form bigger particles. Also, some bixin crystals were present between agglomerates or inside elongated amorphous particles. On the other hand, CRB-Whey microcapsules (Figure 4j) showed spherical particles agglomerating into bigger aggregates. It is worth mentioning that some CRB particles were not encapsulated by whey.

#### 3.4.3. Microscopic Characteristics

**Figure 5:**
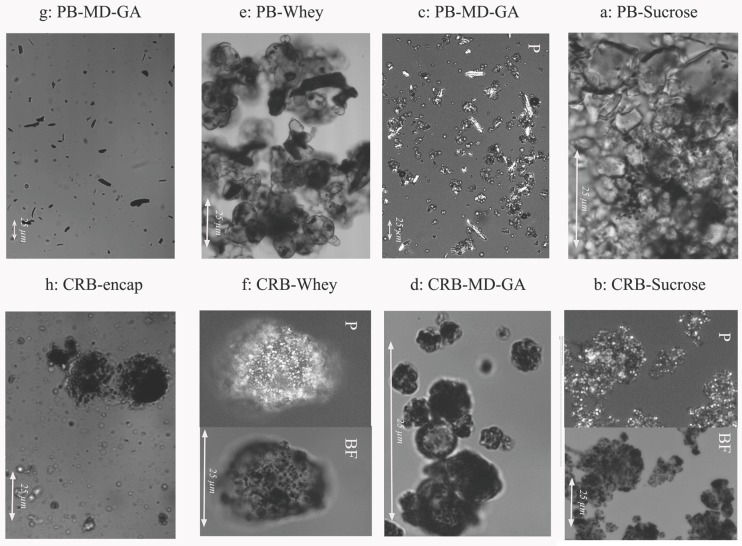
Light microscope images of pure bixin and CRB microcapsules imbibed in MCT oil. (**a**) PB-Sucrose, non-polarized light ×100; (**b**) CRB-Sucrose, non-polarized (left) and polarized light (Right) ×50; (**c**) Bi-MD-GA, polarized light, ×50; (**d**) CRB-MD-GA, non-polarized light, ×100; (**e**) PB-Whey, non-polarized, ×50; (**f**) CRB-Whey, non-polarized (left) and polarized light (Right), ×100; (**g**) PB-MD dispersed in water, non-polarized light, ×20; (**h**) PB-MD dispersed in water, ×50; (**g**,**h**) are representative images of all the water dispersions of spray dried systems containing PB and CRB respectively.

It is possible to differentiate the size of bixin crystals in both sets of microcapsules (PB or CRB) observing the microcapsules using light microscopy (Figure 5). Figure 5a shows bixin crystals that are agglomerated between sucrose crystals without proper microcapsule formation. In CRB-sucrose (Figura 5b) as well as in other CRB microcapsules, a homogeneous dispersion of bixin crystals can be observed inside the microcapsules. When pure bixin crystals are encapsulated with MD (Figure 5c) or whey (Figure 5e), the crystal sizes are much bigger than in CRB-MD (Figure 5d) and CRB-Whey (Figure 5f). 

Figure 5g,h show representative light microscope images of microcapsule water dispersions of the different systems. It can be noticed that there is a big difference between the samples containing PB crystals compared to the ones containing CRB extract. Figure 5g of the PB crystals shows that the encapsulant material is fully dissolved in water and bixin crystals are dispersed in water without any coating. In contrast, Figure 5h of the CRB particles shows bixin coated crystals present in particles with remaining native carbohydrate material regardless of the dissolved encapsulant. Thus, it is shown that the native carbohydrate fraction is remaining, coating the bixin crystals, and keeping them together as a more or less spherical particle or agglomerate. 

### 3.5. Properties of Aqueous Dissolved Microcapsules

#### 3.5.1. Colorimetric Properties

**Figure 6:**
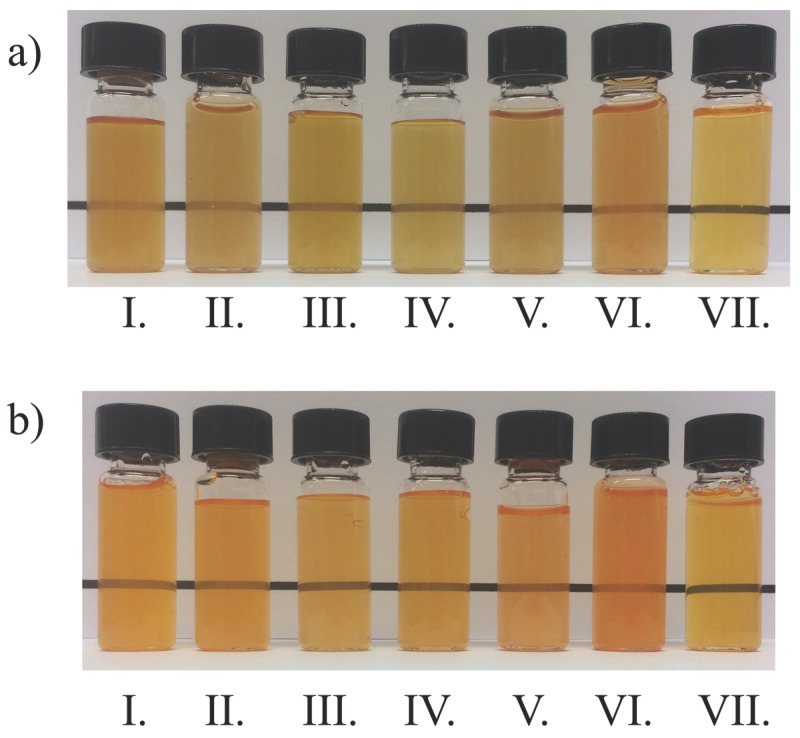
Aqueous dispersions of CRB (**a**) and PB (**b**) microcapsules at 0.3 mg/mL and 1.5 mg/mL respectively at alkaline pH. Aqueous dispersions of CRB and CRB-encap at 0.048 mg/mL. From left to right I: MD, II: MD-GA, III: MD-CMC, IV: MD-Pectin, V: Whey, VI: Sucrose, VII: (**a**) CRB-encap, VII: (**b**) CRB.

The CIE Lab parameters of the microcapsule water solutions/dispersions are shown in Table 3. The PB dispersions had yellower–brownish colors (Figure 6b), while CRB microcapsules gave intense orange colorations (Figure 6a). L values are higher for CRB samples than for PB samples meaning that CBR dispersions have brighter colors as can be compared in Figure 6a,b. In addition, CRB dispersions were less turbid than the samples that contained PB particles. Notice that colorant particle sizes affected the colorimetry of the aqueous dispersions because PB microcapsules needed a concentration five times higher than CRB microcapsules for comparable color intensities. 

**Figure 7:**
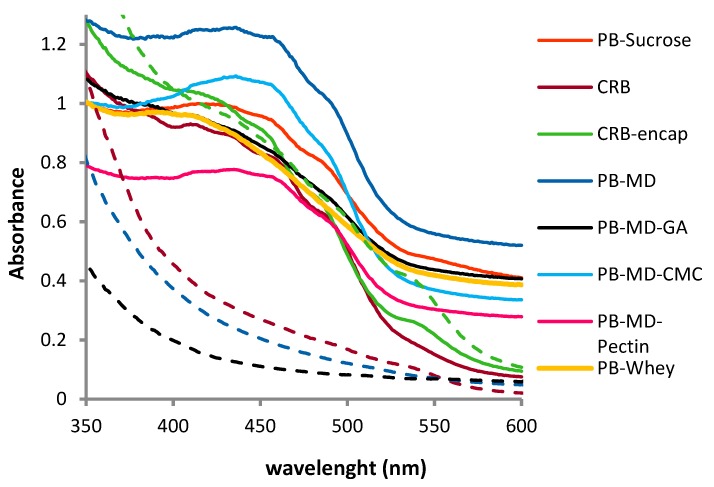
Visible spectra of pure bixin microcapsules aqueous solution/dispersions before and after physical stability tests. At 0 h, concentrations of CRB and CRB-encap were 0.1 mg/mL and 0.048 mg/mL respectively. Visible spectra after physical stability tests were taken of the supernatant of samples prepared at 0.3 mg/mL for CRB and CRB-encap. For all other systems, 1.5 mg/mL was employed. The solid lines show the visible spectra at time 0 h, whereas the dashed line shows the lack of absorbance (characteristic bands) after the colorant particles have settled.

**Figure 8:**
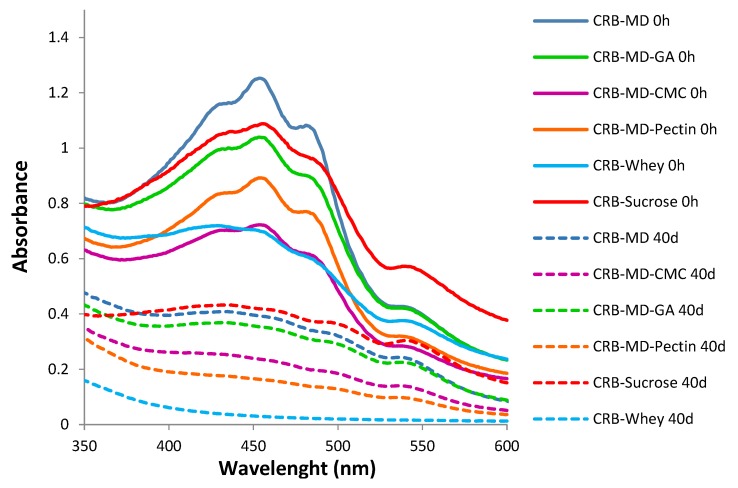
Visible spectra of pure CRB microcapsules aqueous solution/dispersions before and after physical stability tests. Stability tests conducted at 0.3 mg/mL for all systems. The solid lines show the characteristic bands for the CRB systems at 0 h, whereas the dashed lines show the decrease in the absorbance after 40 days. Notice the lack of absorbance for CRB-Whey (dashed line) after 40 days.

The visible spectra of the bixin microcapsules dissolved/dispersed in water and CRB microcapsules aqueous solutions/dispersions are presented in Figure 7 and Figure 8 respectively. It can be noticed that the typical bands in bixin visible spectra at 430, 450 and 480 nm are not present in PB dispersions. On the contrary, these bands are preserved in the CRB samples except for CRB encapsulated together with whey. 

Figure 7 shows the comparison of UV spectra for PB-MD and PB-MD-GA dispersions at day 0 and after 8 days of physical stability tests (Section 3.5.2). The absorbance in the visible range disappears completely as a result of complete sedimentation. It is possible to compare the UV spectrum of CRB microcapsules dispersed in water with their supernatant after 40 days of the physical stability study (Figure 8). There is a decrease in the absorbance and a loss of the characteristic bands. However, the absorbance remains significant and offers an appreciable color. 

#### 3.5.2. Physical Stability of Water Dispersions

**Figure 9:**
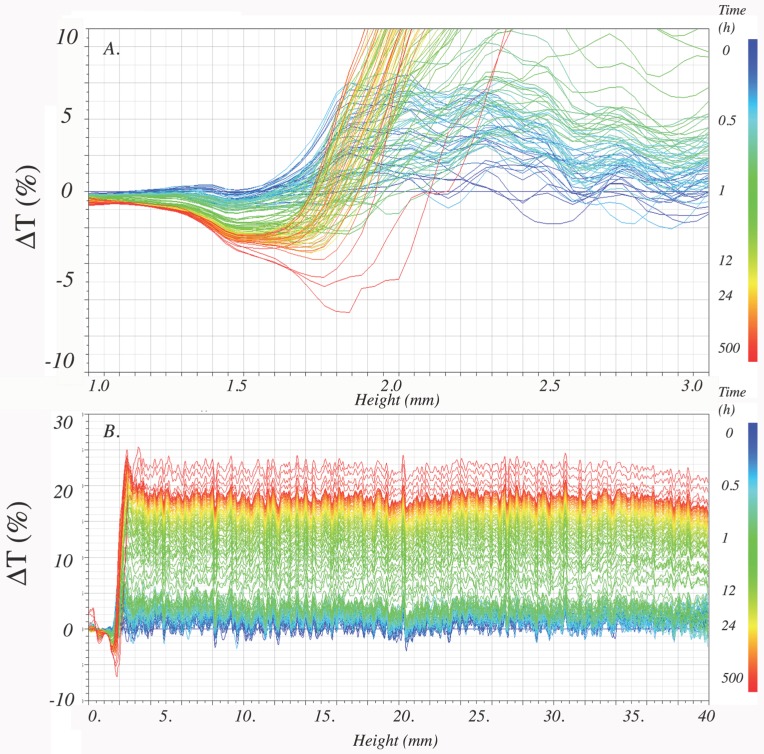
Delta transmittance profile of Bi-MD-Pectin microcapsules dispersed in water obtained over 40 days of storage in sample flasks. Sedimentation is shown in the bottom of the flasks (heights between 1 mm and 3 mm) and flocculation in the middle section of the flasks (heights between 3 mm and 37 mm). A blue line shows proximity to day 0. A red line shows proximity to day 40. (**a**) is an expansion of (**b**) between 1 mm and 3 mm.

After dissolving the microcapsules, the physical (colloidal) stability of the aqueous dispersions of the colorant particles (PB or CRB) was assessed using a Turbiscan equipment. At different intervals, the transmittance of light was scanned through the vertical length of the sample for over 40 days. The difference in transmittance between scans showed sedimentation of the particles and flocculation. A representative profile that belongs to one of the samples, displaying these instability mechanisms, is shown in Figure 9. The transmittance scan profile of most of the samples showed light transmittance above 2% in the bottom region (0–3 mm), and in the middle region of the cell (3–37 mm). Therefore, the delta transmittance profile was analyzed in both regions. In the bottom region of the cell, sedimentation of particles took place, while in the middle region of the cell, flocculation of the particles was observed, which led to further sedimentation (Figure 9). 

Moreover, the sedimentation and/or destabilization profiles were analyzed to obtain a Turbiscan Stability Index–*TSI*. The *TSI* is calculated through the length of the sample, by obtaining the difference between the transmittance profiles at two consecutive time intervals. For a specific time, this parameter can be interpreted as the instant speed at which the system destabilizes. As an example, CRB aqueous dispersion settled readily regardless of the higher bixin content (~50%). Therefore, the higher the Stability Index, the faster the sedimentation or flocculation will happen. 

The Stability Indexes for all samples for different times are presented in Table 4. The most unstable sample was the CRB extract which was not spray dried. This is logical because acid pH neutralizes the charge of the bixin’s carboxylic group and the particles become neutral leading to aggregation followed by sedimentation. 

**Figure 10:**
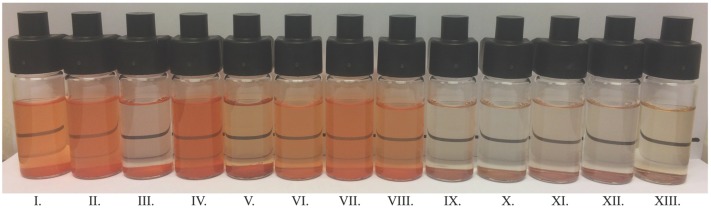
CRB and PB microcapsules dissolved/dispersed in water after 40 days of the physical stability study (0.3 mg/mL). From left to right: I: CRB-MD_,_ II: CRB-Sucrose, III: CRB-Whey, IV: CRB-encap, V: CRB, VI: CRB-MD-CMC, VII: CRB-MD-GA, VIII: CRB-MD-Pectin, IX: PB-MD, X: PB-Whey, XI: PB-Sucrose, XII: PB-MD-GA, XIII: PB-MD-CMC.

There are higher *TSI* values for bixin aqueous dispersions compared to CRB dispersions. After the 8th day, the rate of increment of the *TSI* values decreases and tends to be constant. Besides, the percentage of the volume fraction of the particles for all systems is negligible after 3 h. This may tell us that all the particles or at least the bigger ones would have settled rapidly. Nevertheless, the CRB dispersions kept a noticeable color through 40 days (Figure 10) meaning that smaller particles remained dispersed in these samples ($D_{4,3}^{40\;days}\sim \;0.2-0.8\;\mu m$, Table 2, Section 3.4.1). 

Figure 10 shows the color of the dispersions after 40 days of the stability tests. Although there were small differences in bixin concentration inside the amount of powder used (0.3 mg/mL), the color intensities displayed by the colorant dispersions are similar enough for comparison purposes. Notice that only CRB samples kept and appreciable color. 

### 3.6. Chemical Composition of the Native Carbohydrate Fraction of the CRB

**Figure 11:**
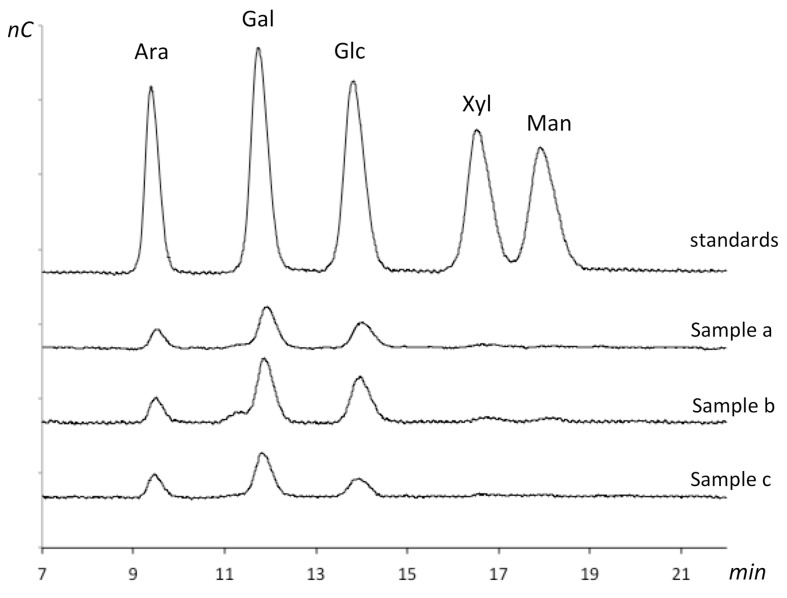
Monosaccharide composition of the hydrolyzed native carbohydrates determined by HPAEC-PAD. The chromatograms show presence of arabinose, galactose and glucose. The standards include arabinose (Ara), galactose (Gal), glucose (Glc), xylose (Xyl) and manose (Man). The samples were analyzed in triplicate (a, b, c).

The HPAEC-PAD analysis (Figure 11) of the hydrolyzed polysaccharides has shown the following monosaccharide composition: arabinose, galactose and glucose. Xylose, mannose and other components present in hemicelluloses were not detected, although, two plausible peaks can be slightly visualized for the retention times of xylose and mannose. 

On the other hand, the enzymatic analysis of the isolated carbohydrate gave significant α-amylase activity and a very weak endo-xylanase activity, which indicates the presence of starch and some xylan (Table 5). Thus, these results are consistent with the monosaccharide composition. 

The relative proportions of arabinose: galactose: glucose detected are 22.2 ± 5.6%:50.2 ± 1.8%:27.6 ± 7.1%. Even though the xylanase activity is very low, there is a previous study that has reported the presence of xylose [7]. It is worth mentioning that the HPAEC-PAD method used in this work, detects only neutral sugars, therefore we cannot discard or confirm the presence of uronic acids or other charged moieties. 

## 4. Discussion

Pure bixin crystals (PB) can be efficiently extracted from the integument of the seeds or may be extracted and purified together with the integument native carbohydrates (CRB). Either of the two colorants can be dispersed in water to be spray-dried with different wall materials. The encapsulation efficiency of the colorant is expected to be determined mainly by the physicochemical characteristics of the wall materials used as encapsulating agents. In that sense, maltodextrin was selected because of its low viscosity at high solution concentrations [17]. Also, CMC, GA and pectin were added due to their encapsulating properties [17]. Alternatively, whey was selected because of its potential binding properties [17]. 

Low molecular weight carbohydrates such as sucrose did not offer protection to pure bixin (PB) since it crystalized together with the colorant after spray drying. However, combining sucrose or lactose with higher molecular weight macromolecules may increase their encapsulating properties. To assess this hypothesis, a comparison of encapsulation efficiencies can be made between CRB-encap and CRB-sucrose, or whey and lactose-whey systems. 

The native carbohydrates fraction of the integument (hemicellulose and starch) may act as a wall material when CRB water dispersions are spray dried. Unfortunately, the low encapsulation efficiency in CRB-encap (i.e., 60%, Figure 4b) suggests that the ratio between the native carbohydrates (encapsulating material) and bixin (colorant) may not be sufficiently high. Interestingly, the addition of sucrose to the CRB dispersion increased the encapsulation efficiency to 92%. The reason could be the low molecular mass of sucrose that allows it to be embedded into the bixin and native carbohydrate microstructure, thereby, making sucrose act as an interacting filler [37]. In another study, the encapsulation of Lutein with MD DE17 and sucrose in a 1:1 relation, gave an encapsulation efficiency of 87% [38]. These comparable results support the idea that high molecular weight macromolecules enable the use of low molecular weight carbohydrates as wall materials. 

Higher molar mass carbohydrates alone, as maltodextrin, are able to form microcapsules. Maltodextrin proved to be suitable as a wall material offering an encapsulation efficiency of 77%. This value is comparable to the one reported by Mercadante, which was 75% [18]. Besides, encapsulation efficiencies for bixin crystals (i.e., 62%) and CRB extract (i.e., 86%) showed that whey protein was not as efficient a wall material as casein (i.e., 90%) [20].

Encapsulation enhancers like gum arabic, CMC and pectin were combined with maltodextrin to increase the encapsulation efficiency of the colorants. Mixtures of MD-CMC1% and MD-GA15% gave the highest encapsulation efficiencies for PB. This could be explained by the emulsifying and film forming properties of CMC [39] and GA [40]. Disappointingly, pectin did not enhance the encapsulation efficiency of maltodextrin as high as CMC and GA did, probably because of the comparable large particle size observed for bixin particles in the PB-MD-Pectin system.

The encapsulation efficiencies of all the wall materials used: MD, MD-CMC1%, MD-GA15%, MD-Pect5% and whey, increased significantly when they were combined with the native carbohydrates of the integument (i.e., >90%). Moreover, the light microscopy images (Figure 5) show that a thin layer remains at the surface of the particles when the added wall material of the CRB microcapsules is dissolved in water. This observation indicates that the native carbohydrate forms the primary layer covering the bixin followed by a secondary layer of wall material. Consequently, the encapsulation efficiency of the microencapsulating system increases. 

**Figure 12:**
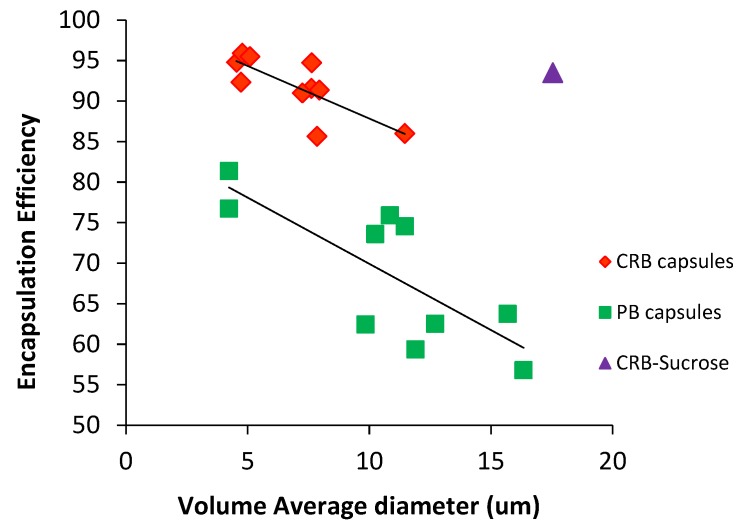
Linear tendency of increasing encapsulation efficiency as a result of decreasing average volume diameter of colorant particles. Each of the encapsulated system replicas was graph against their encapsulation efficiency. Two parallel tendencies are observed: ♦ CRB microcapsules and ■ PB microcapsules.

Another important parameter that affects encapsulation efficiency is the particle size distribution of the colorant dispersion. Our results show a linear tendency of an increase in encapsulation efficiency with the decrease of colorant average volume diameter (Figure 12). This means that a better protection of the colorant will be achieved by decreasing the capsule particle size. This is observed both with pure bixin as well as with CRB microcapsules. This observation is surprising as the colorant surface to volume ratio increases with decreasing size. It should be noted that the spray-dried microcapsules from the laboratory spray-dryer is in a similar size range to colorant particles (core). A possible reason for this could be that efficient encapsulating materials also contribute to particle formation during the spray-drying unit operation. 

Furthermore, Figure 12 (Table 2, Figure 2) shows that bigger colorant particle sizes lower the encapsulation efficiencies of good wall materials such as MD-GA and MD-Pectin in pure bixin systems. However, the excellent encapsulating properties of wall materials such as MD-CMC and the CRB-Sucrose are highlighted since high encapsulation efficiencies were observed even when the colorant had particle sizes above 10 μm. It is worth mentioning that although CRB particles are bigger, the actual crystal size inside CRB agglomerates is smaller. 

The physical stability measurements showed sedimentation and flocculation of pigment particles over time. Also, *TSI* values gave a comparison of the ease with which the particles sediment in each system. Higher stability was observed for CRB aqueous dispersions compared to pure bixin (PB) aqueous dispersions even for similar average volume diameters (~4 μm). This strongly suggests that the presence of the native carbohydrates (hemicellulose and starch) may be stabilizing the CRB particles somehow. 

In the case of CRB-Whey microcapsule, something unexpected was observed. Sweet whey should be dispersible at acidic pH far away from its isoelectric point (IP: 4.5–5.5), but complete sedimentation of pigment particles took place (Table 2, Figure 10). A possible explanation may be the complexation of anionic bixin and cationic whey protein at pH 3. 

After 40 days of the stability study, only the set of CRB dispersions had color remaining and submicron particles (0.3 μm and 0.7 μm). The zeta-potentials values obtained from the remaining particles in the supernatants were around −15 mV despite the low pH (pH around 3). The electrostatic charges producing these zeta-potentials are not enough for stabilizing the CRB submicron particles [20]. Hence, a slow process (>40 days) of flocculation followed by sedimentation takes place due to possible attractive Van der Walls interactions between the CRB particles. The interpretation of flocculation is supported by observations of flocs in the dispersion as well by the presence of sediment with low density at the bottom of the sedimentation cell. 

Furthermore, we hypothesize that spray-drying of the dispersion with the native carbohydrates present is necessary to increase colorant particle stability in aqueous dispersions. We have seen that the stability index for CRB dispersion is much higher than for CRB-encap dispersion (Table 4, Figure 10). In addition, CRB-encap with smaller particles (<7 μm) would have settled faster due to faster flocculation into larger aggregates if flocculation would not have been reduced. Therefore, spray drying the native carbohydrates certainly changes their physicochemical properties increasing the stability of CRB-encap aqueous dispersions.

The chemical characteristics of the native carbohydrate fraction suggest that it is composed mainly by arabinogalactans and starch. The relative composition of arabinose and galactose determined suggest the presence of arabinogalactans. These types of polysaccharide are present in the hemicellulose of seeds in several species [41]. On the other hand, the presence of glucose together with the amylase analysis confirms the presence of starch. The carbohydrate fraction may explain the differences observed in the colorant aqueous dispersibility in basic/acidic conditions as well as before/after spray drying. Some hemicelluloses containing galactose, arabinose and especially xylose, are only soluble/dispersible in alkaline pH [25]. This agrees with the effective CRB water dispersibility at that condition. On the other hand, we speculate that the state of crystallinity of the native starch may prevent the aqueous dispersibility of the particles [42]. However, the ordered structure may be reduced after spray-drying, and thus, it may increase the CRB dispersibility and physical stability. Then, during spray drying, both polysaccharides may interact, thus increasing their dispersibility in acidic aqueous conditions afterwards. 

## 5. Conclusions

Bixin is water dispersible at mild alkaline conditions and will sediment at acidic conditions. Amorphous carbohydrates such as maltodextrin are better wall materials than crystalizing saccharides such as sucrose. Nonetheless, sucrose may act as an encapsulant together with microencapsulation enhancers such as higher molecular weight polysaccharides. Yet, macromolecular encapsulation enhancers such as GA and CMC together with non-crystalline MD are the superior materials for bixin microencapsulation.

The native carbohydrates of the annatto integument enhance the encapsulation efficiency of polysaccharides or protein encapsulants. The presence of starch and arabinogalactans allows the encapsulation of bixin in its native microcrystalline form, resulting in a multilayer structure after spray drying. In addition, particle sizes have an impact on the encapsulation efficiency of bixin crystals. Smaller particle sizes will increase the encapsulation efficiency of wall materials.

Pure bixin microcapsules, without the native carbohydrates, may not be suitable for aqueous products but could improve the shelf life of the colorant, enabling their use in other types of products. 

Spray-drying is an efficient technique for encapsulating bixin dispersions and changing the structural or physicochemical properties of the native carbohydrates to obtain water dispersible formulations that have good colorimetry and physical stability even at acidic pH for at least 40 days. Flocculation and sedimentation over time are not completely avoided due to large particle sizes, neutralization of bixin and aggregation of particles. Nevertheless, these water dispersions have potential in their use in aqueous products such as sodas, juices, candy preparations, etc. Our results show the possibility to increase the efficiency of the microencapsulation process of natural food colorants, a possible reduction in production costs, and they allow for further research developing new water dispersible formulations with natural colorants.

## Figures and Tables

**Table 1:** Initial compositions of the total solids for each prepared system prior spray drying.

System	Bixin	Native Carbohydrates	Sucrose	MD	Whey	CMC	Pectin	GA	Na_2_CO_3_
PB-Sucrose	9.7%		87.6%						2.6%
PB-MD	9.7%			87.6%					2.6%
PB-MD-GA	9.7%			73.0%				14.6%	2.6%
PB-MD-CMC	9.7%			86.7%		1.0%			2.6%
PB-MD-Pectin	9.7%			82.8%			4.9%		2.6%
PB-Whey	9.7%				87.6%				2.6%
CRB-Sucrose	11.9%	11.9%	71.3%						4.9%
CRB-MD	11.9%	11.9%		71.3%					4.9%
CRB-MD-GA	11.9%	11.9%		57.0%				14.3%	4.9%
CRB-MD-CMC	11.9%	11.9%		70.3%		1.0%			4.9%
CRB-MD-Pectin	11.9%	11.9%		66.5%			4.8%		4.9%
CRB-Whey	11.9%	11.9%			71.2%				5.1%
CRB	50%	50%							

MD: Maltodextrine, GA: Gum arabic, CMC: Carbocymethylcellulose, CRB: Native carbohydrate rich bixin (See Appendix A).

**Table 2:** Total bixin (T_B_) contents and water activity (W_a_) of spray dried powders, volume weighted average diameter of the microcapsules dissolved/dispersed in water after spray drying $D_{4,3}^{0\;days}$, and the one of their supernatants after 40 days of the physical stability test ($D_{4,3}^{40\;days}$). (--: not measured, nd: not detected, (1) & (2): Replicates 1 and 2).

Sample	T_B_ (%)	W_a_	$D_{4,3}^{0\;days}$		$D_{4,3}^{40\;days}$	Sample	T_B_ (%)	W_a_	$D_{4,3}^{0\;days}$		$D_{4,3}^{40\;days}$
CRB *	36.0 ± 1.60	--	(1)	74.5	nd	CRB-encap	49.3 ± 2.14	0.41	(1)	8.86	0.19
			(2)	42.4	--				(2)	6.57	--
PB-Sucrose	6.2 ± 0.41	0.37	(1)	7.44	nd	CRB-Sucrose	8.46 ± 0.11	0.30	(1)	7.62	0.23
			(2)	6.45	--				(2)	17.54	--
PB-MD	8.8 ± 1.31	0.39	(1)	10.25	nd	CRB-MD	9.62 ± 0.58	0.27	(1)	4.54	0.31
			(2)	4.23	--				(2)	4.72	--
PB-MD-GA	8.4 ± 0.62	0.38	(1)	15.69	nd	CRB-MD-GA	9.63 ± 0.23	0.29	(1)	4.78	0.25
			(2)	4.23	--				(2)	5.09	--
PB-MD-CMC	6.5 ± 1.56	0.41	(1)	11.46	nd	CRB-MD-CMC	7.34 ± 0.12	0.28	(1)	7.94	0.24
			(2)	10.85	--				(2)	--	--
PB-MD-Pectin	7.4 ± 0.29	0.38	(1)	16.34	nd	CRB-MD-Pectin	7.79 ± 0.25	0.33	(1)	7.24	0.85
			(2)	11.90	--				(2)	7.63	--
PB-Whey	7.5 ± 0.79	0.34	(1)	9.84	nd	CRB-Whey	6.62 ± 0.16	0.30	(1)	7.85	nd
			(2)	12.71	--				(2)	11.46	--

* Total bixin varies between 36 and 50%. The particle size varies as well in the range of 40–80 µm depending on the grinding. The result is an example for a particular batch.

**Table 3:** CIE Lab parameters a, b, L, and turbidity (t) measurements of water dispersions containing 1.5 mg/mL of PB microcapsules, 0.3 mg/mL of CRB microcapsules, or 0.048 mg/mL of CRB and CRB-encap.

Sample	Colorimetry			t at 600 nm
	a	b	L	
CRB	54.29	10.50	50.34	--
CRB-encap	52.77	13.19	66.24	0.22
PB-Sucrose	45.01	12.07	46.13	0.41
CRB-Sucrose	41.90	20.31	56.47	0.38
PB-MD	48.06	10.67	53.70	0.52
CRB-MD	44.00	19.38	72.62	0.23
PB-MD-GA	48.98	8.15	40.29	0.41
CRB-MD-GA	43.40	18.50	67.00	0.23
PB-MD-CMC	5.63	9.30	58.81	0.34
CRB-MD-CMC	53.20	11.10	44.68	0.17
PB-MD-Pectin	53.63	6.28	40.46	0.28
CRB-MD-Pectin	47.69	14.21	59.06	0.19
PB-Whey	48.62	8.05	39.09	0.39
CRB-Whey	48.21	13.89	39.72	0.24

**Table 4:** Stability Indexes of microcapsule water dispersions at different periods of time. (--: not measured, (1) & (2): Replicates 1 and 2). Replicates (1) and (2) had a concentration of 0.3 mg/mL and 1.5 mg/mL of microcapsule powder respectively.

Sample		*TSI*				
	R	20 min	2 days	8 days	23 days	40 days
CRB	(1)	3.7	55.7	63.0	66.6	67.8
	(2)	7.8	38.4	42.4	55.5	72.0
CRB-encap	(1)	2.2	22.8	28.9	32.4	33.5
PB-Sucrose	(1)	1.1	15.7	22.2	25.1	--
CRB-Sucrose	(1)	0.7	17.5	25.7	34.2	39.3
PB-MD	(1)	0.9	23.0	31.1	34.7	--
	(2)	1.0	39.4	56.0	67.2	--
CRB-MD	(1)	1.3	17.2	22.2	27.2	30.3
PB-MD-GA	(1)	1.2	22.5	27.9	29.8	--
	(2)	2.0	22.8	48.8	65.3	--
CRB-MD-GA	(1)	1.0	15.5	20.5	24.8	28.4
PB-MD-CMC	(1)	1.9	24.3	27.1	29.1	--
CRB-MD-CMC	(1)	1.6	18.1	22.9	26.6	28.7
PB-MD-Pectin	(1)	1.9	21.0	23.6	--	--
CRB-MD-Pectin	(1)	1.8	20.1	24.9	29.3	32.6
PB-Whey	(1)	1.9	25.0	28.6	31.1	--
CRB-Whey	(1)	0.8	48.0	48.7	49.7	49.9

**Table 5:** Enzymatic activities on the native carbohydrate fraction (1% *w*/*v*) after 14 h of incubation, determined in absorbance units by DNS assay [31]. (nd = no detected activity).

Enzyme	Activity (Abs/14 h)
*endo*-arabinase	nd
α-amylase	2.615
Cellulase	nd
xylanase	0.038

## Supplementary Materials

The following are available online at https://www.mdpi.com/2304-8158/8/3/108/s1, encapsulation efficiencies calculations: Encapsulation Efficiency – Supplementary Data.

## Abbreviations

A list of abbreviations is given below:

PB	Pure Bixin
CRB	Carbohydrate Rich Bixin prior spray drying
CRB-encap	Carbohydrate Rich Bixin encapsulated (spray dried CRB)
PB-Sucrose	Pure Bixin-Sucrose
PB-MD	Pure Bixin-Maltodextrine
PB-MD-GA	Pure Bixin-Maltodextrine-Gum Arabic
PB-MD-CMC	Pure Bixin-Maltodextrine-Carboxylmethylcecllulose
PB-MD-Pectin	Pure Bixin-Maltodextrine-Pectin
PB-Whey	Pure Bixin-Whey
CRB-Sucrose	Carbohydrate Rich Bixin-Sucrose
CRB-MD	Carbohydrate Rich Bixin-Maltodextrine
CRB-MD-GA	Carbohydrate Rich Bixin-Maltodextrine-Gum Arabic
CRB-MD-CMC	Carbohydrate Rich Bixin-Maltodextrine-Carboxyl Methyl Cellulose
CRB-MD-Pectin	Carbohydrate Rich Bixin-Maltodextrine-Pectine
CRB-Whey	Carbohydrate Rich Bixin-Maltodextrine-Whey
CRB	Carbohydrate Rich Bixin
HPAEC-PAD	High Performance Anion-Exchange Chromatography Coupled with Pulsed Electrochemical Detection
